# The needs of key-stakeholders for evaluating client’s experienced quality of home care: a qualitative approach

**DOI:** 10.1186/s41687-020-00260-3

**Published:** 2020-11-10

**Authors:** Roy Haex, Theresa Thoma-Lürken, Sandra Zwakhalen, Anna Beurskens

**Affiliations:** 1 CAPHRI Care and Public Health Research Institute, Department of Health Services Research, Living Lab on Ageing and Long-Term Care, Maastricht University, Maastricht, the Netherlands; 2 Faculty of Health, Zuyd University of Applied Sciences, Heerlen, the Netherlands; 3 CAPHRI Care and Public Health Research Institute, Department of Family Medicine, Maastricht University, Maastricht, the Netherlands

**Keywords:** Care relationship, Experienced quality, Home care, Nursing, Older people, Informal caregiving, Quality of care, Needs assessment

## Abstract

**Background:**

To optimize home care, it is essential to determine how care recipients experience quality of care. Traditionally, quality of care is measured with normative quality indicators such as safety, efficiency, or prevalence rates such as falls. The growing interest for qualitative patient-reported experience measures in home care requires insight into the needs of care receivers, providers, and organizations as key-stakeholders. Each stakeholder has their own needs that are important to communicate and use to conduct thorough comparisons before implementing new experience measures. This study aims to understand the needs of clients, formal/informal caregivers, and managers/policy officers in measuring client’s experienced quality of care in home care.

**Methods:**

Four focus group interviews and 25 semi-structured interviews with key-stakeholders were conducted and analyzed by means of content analysis. The value-proposition canvas was used as a thematic framework to explore the purpose of experience quality of care measures and related pains and gains.

**Results:**

There were two main purposes for measuring experienced quality of care: first improving the primary care process of individual clients and second for learning and improving in home care team. Using experienced quality of care measures for external accountability and transparency on an organizational or national level were considered less relevant. Among others, participants described not having time and no clear procedure for conducting an evaluation as a pain of the current methods used to evaluate perceived quality of home care. As gains they put forward the ability to informally evaluate experiences during care delivery and to openly discuss complaints with a familiar caregiver.

**Conclusions:**

This study advocates that home care organizations should be aware of the goal of quality of care measures. They should consider selecting experienced quality of care measures mainly for improving primary care processes of individual clients. The results also underline the relevance of adopting next to quantitative evaluations, more narrative evaluation methods which support communicating openly on care experiences, leading to concrete point-of-improvement. The findings of this study can serve as a guide for both the development or selection of adequate methods, from the perspectives of key-stakeholders, in assessing experienced quality in home care.

**Supplementary Information:**

The online version contains supplementary material available at 10.1186/s41687-020-00260-3.

## Background

In the European Union, the percentage of people aged 80 and older is expected to increase from 5.4% in 2016 to 12.5% in 2070 [1]. With an aging population, there is an increasing need for home care services to facilitate aging in place [2, 3]. In the Netherlands, the percentage of people receiving long-term care at home has increased by 23% in 2018 compared to 2015 [4]. To optimize home care, it is essential to determine how clients experience quality of care (QoC). Traditionally, QoC has been defined using criteria such as safety, efficiency, or effectivity or prevalence rates of care problems, such as falls and the use of physical restraints [5, 6]. These criteria can be measured in home care using existing quantitative instruments such as the National Prevalence Measurement of Quality of Care [7]. Besides measuring criteria from a quantitative point of view, it is essential to understand the individual needs of clients and their experiences with home care services to assess the experienced QoC [8–10]. Although long-term home care is traditionally provided to clients aged 65 and above, there is a proportion of clients under 65 who, for instance, receive short-term home care while recovering from hospital care and may have differing preferences communicating care needs [11, 12]. Furthermore, it is increasingly important to include informal and formal caregivers to understand and interpret the experienced QoC in the home care context since quality is achieved during interactions between caregiver and receiver [13–15]. Existing measures for experienced QoC in home care, such as the Net Promotor Score (NPS) or rating caregivers online (e.g. via a website named ZorgKaart Nederland), focus mainly on satisfaction which is defined as a subjective evaluation of the gap between a client’s care expectations and experiences [16–18]. Furthermore, in the Netherlands QoC in home care is often evaluated yearly or half-yearly by formal conversations between district nurses and clients, in addition to more informal evaluations by direct caregivers during care provision [19]. However, no obligatory or clearly defined format and structure have been established for these evaluations.

In 2018, a new national quality framework was released in the Netherlands stating the importance of utilizing patient-reported experience measures (PREMs) to gain insight into the experienced QoC in home care [19]. However, before deciding how experienced QoC should be measured it is important to define why it should be measured (e.g. the goal), who will use it (e.g. the key-stakeholders) and determine the context surrounding the method that is being applied (e.g. when, where, and by whom) [20]. For example, a nurse may choose a tool to find specific points-of-improvement in the daily care routine using different requirements regarding eligible instruments compared to a policy officer’s goal to publicly report the organization’s care quality. It is therefore important to distinguish three different goals in evaluating experienced QoC. The first goal is to provide insight into clients’ experiences so the primary care process can improve. The second goal is to assist care teams in creating an environment that facilitates learning from and improving the experienced QoC. The final goal is to use the evaluations on an organizational or national level for external accountability and transparency [20].

Involving key-stakeholders such as clients, (in) formal caregivers, and managers/policy officers as active partners is crucial to gain insights in the needs for measuring experienced QoC in home care. Doing so ensures that the chosen methods have greater value for stakeholders in both the direct care process and organization [21, 22]. It is expected that each stakeholder has their own needs, which can either facilitate or hinder the implementation of a new method [23]. As a consequence, by incorporating these needs in determining a method for experienced QoC, a better connection can be made to the organizational current workflow and individual home care processes [24]. In addition to stakeholders in the primary care process (clients, informal, and formal caregivers), care organizations can play a facilitating role in implementing interventions such as measures for experienced QoC [23]. Managers and policy officers of care organizations make decisions regarding allocating resources for QoC measures to be incorporated in all layers of the organization [21, 25]. The needs of clients, informal caregivers, formal caregivers, and managers/policy officers as key-stakeholders to evaluate experienced quality in home care are currently unknown. Therefore, the aim of this study is to gain insight into the needs of clients, formal and informal caregivers, and managers/policy officers in measuring client’s experienced QoC in home care.

## Methods

### Design

This qualitative study followed a descriptive design in which a needs assessment was conducted using principles of the Value Proposition Canvas (VPC) for structuring and analyzing the needs of clients, formal and informal caregivers, and managers/policy officers [26]. The VPC has been applied in healthcare to involve stakeholders in improving the value of new instruments, services, and products [24]. The VPC is developed to match the needs of key-stakeholders with the value proposition of the method, and thus achieve a problem-solution fit [27]. The VPC differentiates between determining the *customer-profile* (who are they) and *value map* (which features are of value). This study adopted principles of VPC and an underlying human-centered design approach helps to understand key-stakeholders’ current situation in measuring QoC and allows for identifying a method fitting their needs best.

### Setting

The research took place between July 2017 and May 2019 as part of a larger study, within three publicly funded, team-based home care organizations in the southern part of the Netherlands.

### Participants and data collection

Individual and focus group interviews were used for data collection. Convenience sampling was used to recruit participants for each group of key-stakeholders, while striving for a balance in distribution between the different groups of stakeholders. A total of 25 semi-structured interviews and four focus groups with 15 participants were conducted. Focus group interviews were used, in addition to individual interviews, since it helps participants to identify, share, and clarify their views [28]. It is expected that experiences and opinions are shared that might not emerge during individual interviews [29]. A topic list with exemplary questions from both the individual and focus group interviews is included in the Additional file 1.

#### Individual interviews

For the individual interviews, clients, informal caregivers, formal caregivers, and managers/policy officers were recruited from three home care organizations in the Living Lab in Aging and Long-Term Care South Limburg [30]. Home care clients were eligible to participate if they were receiving long-term home care based on at least one chronic condition and were both mentally and physically able to participate according to their district nurse. Informal caregivers were eligible if they provided care for home care clients. Both clients and their informal caregivers were informed by their formal caregiver first and asked for permission to be contacted by the research team. Formal caregivers were eligible if they currently worked in home care as a district nurse, nurse assistant, nursing aide, or dementia case manager. Dementia case managers are professionals supporting and advising people with dementia and their family in the diagnostic phase and coordination of care [31]. In addition, the organization’s district-managers and policy officers were eligible if they were working in long-term home care. Both formal caregivers and managers/policy officers were contacted by either mail or telephone and invited to participate. Individual semi-structured interviews were scheduled at a preferred location of the participant (either at home, the care organization, or at the university). If necessary, the informal caregiver could support the client in the interview (e.g. if a client had trouble speaking clearly) but was asked to not actively engage in the conversation. The planned duration for the individual interviews was 1 hour.

#### Focus group interviews

Four focus group interviews were conducted to gain insight into the current methods and needs of measuring experienced QoC in home care. For two focus group interviews, clients and informal and formal caregivers employed in a home care organization were eligible and invited. However, two informal caregivers and one client could not attend this focus group because of personal circumstances for which one additional focus group was organized. In addition, one focus group interview was conducted with managers/policy officers in order to include the perspectives from the participating organizations. This was done to prevent any communicating difficulties as a result of status differences between participants (caregivers – managers/policy officers) or by discussing technical terms beyond the scope of the direct caregiving process (e.g. legislative, regulatory or organizational requirements) [32]. The discussion leader (RH) took field notes to log the context of the focus group interviews and to provide meaning to the reported needs. Focus group interviews were scheduled to last around 1 hour.

### Data analysis

Both the individual and focus group interviews were audiotaped and transcribed verbatim. The data from both the individual and focus group interviews were merged and analyzed using MAXQDA Standard 2018, following principles of directed content analysis [33]. In the deductive analysis, the VPC was used as a thematic framework to categorize key-stakeholders’ needs into goals, pains or drawbacks of current evaluation methods and (desired) gains or benefits as a priori themes (see Table 1 for operational definitions) [26].

**Table 1:** A priori themes and operational definitions, based on the VPC [27]

A priori themes	Operational definition
*Goals*	Purposes or tasks that key-stakeholders strive to satisfy by measuring experienced QoC. These were categorized in the following three categories: 1) understand and improve the primary care process for individual clients; 2) learn and improve the performance of home care teams based on the outcomes of quality measurements; 3) use outcomes for external accountability, transparency, and generally improve organizational service provision [20].
*Pains*	Drawbacks of current methods to measure QoC, negative emotions, undesired situations, or risks key-stakeholders have experienced before, during, or after fulfilling the goals.
*Gains*	Benefits of current methods and desires in measuring QoC.

New categories and sub-categories were identified from both the pains and gains by means of inductive analysis. Using condensation, the categorized data was shortened while preserving the core meaning [34]. Next, the condensed data was interpreted using a higher logical level, also known as abstraction. This was followed by axial coding in which the individual pains and gains were categorized, and sub-categories emerged through careful examination and constant comparison by two researchers independently (RH and TTL). In case of disagreements, the researchers discussed the (sub) categories to reach a consensus.

### Rigour

Several strategies recommended by Korstjens and Moser [35] were used to meet the criteria of credibility, transferability, dependability, confirmability, and reflexivity, thereby strengthening the trustworthiness of this study [36]. To increase credibility, the results were presented during two group meetings with six participants to verify correct interpretation and completeness of the results. To enhance transferability, a detailed description was made on the context of the research, setting, sample, demographics, and exemplary quotes. Furthermore, a detailed codebook was made to keep track of all data-driven codes (categories and sub-categories) during the analysis.

## Results

A total of 25 participants took part in the individual interviews and 15 participants took part in four focus group interviews. The mean duration for individual interviews and focus group interviews were 55 min and 63 min respectively. Table 2 provides information about the demographics of the participants and composition of the focus group interviews. Outcomes of the individual and focus group interviews displayed different goals, pains, and gains for measuring experienced quality in home care.

**Table 2:** Demographics

		N	Sex (male, female)	Median age (range)
Individual interviews (*N* = 25)				
Role	Client	6	2 male, 4 female	85.5 (19)
	Informal caregiver	6	3 male, 3 female	72 (27)
	Nurse aide/assistant	2	2 female	46 (30)
	Dementia case manager	1	1 male	53
	Registered nurse	1	1 female	63
	District nurse	6	1 male, 5 female	25.5 (30)
	Manager/Policy officer	3	3 female	54 (27)
Focus group 1 (*N* = 7)				
Role	Client	2	1 male, 1 female	80.5 (8)
	Informal caregiver	3	1 male, 2 female	77 (16)
	District nurse	2	2 female	33 (14)
Focus group interview 2 (*N* = 2)				
Role	Nurse aid/assistant	1	1 female	61
	Registered nurse	1	1 female	64
Focus group interview 3 (*N* = 3)				
Role	Client	1	1 female	86
	Informal caregiver	2	1 male, 1 female	76.5 (17)
Focus group interview 4 (*N* = 3)				
Role	Manager/Policy officer	3	1 male, 2 female	54 (21)

### Goals in measuring experienced quality (why)

All stakeholders mentioned goals related to at least one of the three main goals for evaluating experienced QoC. The first goal of “understanding and improving the primary care process for individual clients” was mentioned by all key-stakeholders. Clients focused mostly on problem-solving when being dissatisfied to improve care provision. One client mentioned that “*if I receive care from a specific caregiver and I am dissatisfied about the care, I would make this clear*.” Informal caregivers tried to provide direct feedback to the formal caregiver when possible in order to improve the primary care process. Formal caregivers indicated that they strove to remain critical and wanted to have a clear/genuine picture of the client’s and informal caregiver’s experienced QoC. Managers/policy officers preferred obtaining structural feedback of the client’s fulfilled and unfulfilled needs and experienced QoC for each area of interest so care provision could be improved.

The second goal of “learning from and improving the performance of home care teams based on the outcomes of quality measurements” was mentioned by formal caregivers, managers/policy officers and informal caregivers. The importance to create awareness for evaluating experienced quality in care teams from an organizational perspective was mentioned by both informal and formal caregivers. This awareness would enable caregivers to work toward concrete care improvements as a team. For instance, one formal caregiver mentioned, regarding evaluating experienced quality, that “*I think you can use it [experienced quality of care assessments] to steer the care process and also with your team or with employees or with the entire organization you can look at which points scored less and how and when will we tackle them*”*.* Managers/policy officers aimed to generally improve care quality by discovering concrete points-of-improvement for care teams. This resulted in formulating appropriate actions to learn and improve for both individual teams and the organization as a whole.

The last goal of “using outcomes for external accountability, transparency, and generally improving organizational service provision” was only noted by managers/policy officers. Use of a yearly mandatory measure for experienced quality was mentioned to meet the requirements of external accountability, as well as providing information on the organization’s website for transparency toward current and possible new clients.

### Pains and gains in measuring experienced quality

The described pains and gains were categorized in the following categories: *when to evaluate*, *who should evaluate*, *how to evaluate*, *what motivates one to evaluate, what to do with outcomes,* and *prerequisites for evaluating*. Next, the results for each of the categories are presented.

#### When to evaluate

In deciding when to evaluate QoC (see Table 3 for an overview), participants in general would like to have more evaluations compared to the current yearly or half-yearly evaluations. Additionally, more flexibility in evaluation frequency was desired, based on the client’s condition. Both clients and informal caregivers preferred to have more opportunities to initiate an evaluation more proactively, thus preventing the escalation of an unsatisfactory experienced QoC. Moreover, formal caregivers needed clarification to plan mandatory evaluations with a set interval, given the goal of evaluating (e.g. once every 6 months).

**Table 3:** Identified pains and (desired) gains for ‘when to evaluate’

Category	Pains	Gains
When to evaluate	▪ Too few evaluation moments, especially during complex care (I,P) ▪ Difficult finding appropriate moment to evaluate (I) ▪ Not knowing when to evaluate (F)	▪ Taking opportunity for evaluating when required (C,I,F) ▪ Starting evaluation based on signals from other caregivers (F) ▪ Starting evaluation based on own experiences as caregiver (F) ***Desired gains*** ▪ *Reminder of the next evaluation (F)* ▪ *Mandatory for each new client (F)* ▪ *Evaluate at set intervals (F)*

Mentioned by *C* Clients, *I* Informal caregivers, *F* formal caregivers, *P* managers/policy officers

Current pains regarding when to evaluate QoC were the few evaluation moments, specifically for clients who receive more complex home care. Moreover, pains included not knowing when to formally evaluate the experienced QoC and when it is most effective to evaluate the care process. Furthermore, informal caregivers often had difficulties finding an appropriate moment during the care provision to evaluate QoC. For instance, one informal caregiver mentioned that there was “*No room to evaluate the client, this comes in last place*.” However, some participants mentioned as a gain that the initiative to evaluate QoC is taken whenever it seems required (e.g. when an informal caregiver is dissatisfied about the care provision for the client). Furthermore, formal caregivers preferred to either initiate an evaluation based on signals from other caregivers or based on their own experiences with the client. Moreover, a formal caregiver indicated that it would be desirable to clarify the frequency of evaluations.

#### Who should evaluate

In determining who should conduct quality evaluations (see Table 4 for an overview), more flexibility for direct caregivers to formally evaluate experienced QoC was desired since this was currently not their role. However, taking into account the importance and fragility of care relationships while evaluating was brought forward. Appointing an external person to evaluate more sensitive topics was seen as a possible solution for this. Including the informal caregiver’s perspective in evaluations is also needed. Furthermore, clearly indicating whom is appointed to evaluate experienced QoC is needed by informal caregivers. They also desired for evaluations to be conducted by the same evaluator(s).

**Table 4:** Identified pains and (desired) gains for ‘who should evaluate’

Category	Pains	Gains
Who should evaluate	▪ Direct caregiver lacks role to formally evaluate (F,P) ▪ Experienced difficulties if evaluation was conducted by direct (influencing existing relationship on responses) vs indirect caregiver (difficulties interpreting responses client) (F,P) ▪ Missing perspective of informal caregiver (F,P) ▪ Difficulties identifying evaluator (I)	▪ Sufficient availability of caregivers to discuss care experiences (I) ▪ Client can decide if family is included (F) **Desired gains** ▪ *Direct* caregiver *instead of district nurse (C,F)* ▪ *Someone with care expertise but not a* caregiver *(I,P)* ▪ *Evaluation to be conducted by direct caregiver so it is relevant to their own experience, they can learn directly and feel responsible for their own clients (F,P)* ▪ *More colleagues should have evaluation discussions (F,P)* ▪ *Overarching contact beyond direct care (I)* ▪ *Same person (I)*

Mentioned by *C* Clients, *I* Informal caregivers, *F* formal caregivers, *P* managers/policy officers

A current pain was that direct caregivers do not have the formal role to formally evaluate experienced QoC. Currently, the district nurses have the formal responsibility to evaluate experienced quality of care every 6 months and not the nurses or nurse aids who provide everyday care. One formal caregiver mentioned, “*Nurse aides have no formal role in evaluating; informal evaluations do take place … [I] experience a big difference between the two district nurses with regard to evaluating care, nurse aides should be more involved*.” The importance of care relationships between the client and formal caregiver was mentioned as well as the difficulties, due to a dependency in relationship, this poses in evaluating QoC. For instance, one policy officer stated, “*You are still dependent within the care relationship … If I am your caregiver, then I should not ask you about it [experienced quality of care]*.”

The size of care teams and the sufficient availability of caregivers was mentioned as a possible gain in evaluating QoC. The size of care teams provided informal caregivers the flexibility to choose whomever they would prefer to informally discuss care experiences. Involving family during evaluations was seen as important by formal caregivers, although whether to involve them should be decided by the client. Most clients and formal caregivers wished to evaluate QoC with direct caregivers. While informal caregivers and policy makers noted flexibility was viewed as a gain by some, formal caregivers mentioned it as a pain when sensitive topics had to be discussed in a formal evaluation. This is also related to the desire to involve an external person with care expertise when formally evaluating, facilitating a more open conversation. For instance, one informal caregiver mentioned that “*If there are problems then you should be able to discuss these with whoever [formal caregiver] is coming to your home, but if there are difficulties with the whole [care process] … .then you should be able to address them to someone else*.” The desire for more colleagues to conduct evaluations and provide possible insights to direct caregivers was seen as important. One informal caregiver elaborated on these insights and the potential dilemma of an evaluation by a direct caregiver or external person: “*People who have the evaluation conversations themselves about their own clients can learn a lot immediately … how honest is that person to you when you hear the information from the client*.”

#### How to evaluate

Regarding how to evaluate QoC (see Table 5 for an overview), there is a need from participants for an evaluation method which requires minimal skills and time to analyze and document outcomes. Methods that are suitable to the ongoing care processes and existing care relationships were seen as important by most. In addition, participants preferred to be more aware of existing evaluation methods, favoring methods based on conversations (e.g. narratives) in which the experiences are evaluated.

**Table 5:** Identified pains and (desired) gains for ‘how to evaluate’

Category	Pains	Gains
How to evaluate	▪ Difficulties finding time to use, analyze, and document existing evaluation methods (I,F,P) ▪ Not suitable with ongoing care process, creating worry that client will be treated as new and unknown (F,P) ▪ Missing supportive methods to evaluate (F,P) ▪ Physical properties of evaluation on paper (F) ▪ Provide only snapshot of client (P) ▪ Current questions asked are too broad, leaves room for too many interpretations (P)	▪ Patient file as starting point evaluation (F) ▪ Room for humor during evaluation (F) ▪ Adjusting way of evaluating to understanding client (F) **Desired gains** ▪ *Sharing experiences during conversation (C,F)* ▪ *Discussing multiple subjects and look beyond standard quality indicators (F,P)* ▪ *Sharing expectations of care services (F)* ▪ *Ability to evaluate anonymously by mail (I)* ▪ *Visually supporting method to evaluate (F)* ▪ *Interactive application (F)* ▪ *Measurement should connect to existing ICT platforms (P)*

Mentioned by *C* Clients, *I* Informal caregivers, *F* formal caregivers, *P* managers/policy officers

Most participants mentioned pains in finding time to use, analyze, and document existing evaluation methods. They also worried that existing methods do not fit within the current care processes, possibly resulting in treating the client as a new and unknown individual. Other pains that were highlighted were the common physical properties of paper-based evaluations (e.g. possibility of becoming lost, damages easily), not evaluating QoC continuously, and asking questions during evaluation which leave too much room for interpretation errors.

The gains of the current methods to evaluate QoC were the access to existing patient files as a starting point for care evaluations. Humor during evaluations and flexibility in adjusting to the client’s understanding were seen as important. Most clients and formal caregivers wished for care experiences to be shared more during conversations, looking beyond discussing standard quality indicators. Furthermore, participants mentioned that the evaluation tool should include sharing expectations of home care services, to prevent unrealistic expectations of evaluation outcomes by both clients and informal caregivers. Furthermore, the desired evaluation tool functionalities were cited such as sharing experiences anonymously by mail, obtaining visual supporting methods (e.g. a card containing images of relevant QoC topics), stimulating a more interactive evaluation by means of a digital application, and connecting to the existing organizational ICT platform (e.g. online care plan, OMAHA system, etc.).

#### What motivates one to evaluate

In determining what motivates one to evaluate (see Table 6 for an overview), it is desired that evaluations should be framed as a positive element in the care process. Allowing the possibility to evaluate anonymously was also mentioned by participants. It is believed that this would facilitate clients to honestly reflect on their care experiences and experienced QoC. In addition, participants mentioned the importance of motivating formal caregivers to incorporate evaluations in the daily care process, creating a shared feeling of ownership for the evaluation method and sharing experiences in care teams. Furthermore, participants noted that evaluations should be perceived as non-intrusive by clients and clients should feel more involved in the care process.

**Table 6:** Identified pains and (desired) gains ‘what motivates to evaluate’

Category	Pains	Gains
What motivates one to evaluate	▪ Evaluating is seen as complaining (C,I,F) ▪ Experienced lack of motivation to evaluate (F,P) ▪ Experienced duplications when formally evaluating (F,P) ▪ Difficulties expressing experiences and being sincere (I,F) ▪ Feel not being heard by caregiver (C) ▪ Feel burdened discussing organizational issues (I) ▪ Being on your own as caregiver in home environment (F) ▪ Experienced having to be more involved with their own clients in sheltered housing vs. community (P) ▪ Not part of care process or professional community (P)	▪ Feels good to talk about care experience (C) ▪ Sharing care experiences is good for involving client in care process (F) ▪ Show understanding and taking changes seriously (F) ***Desired gains*** ▪ *Evaluate anonymously (I,F)* ▪ *Team ownership and bottom-up support for tools (F,P)* ▪ *Build toward relationship with evaluator (I)* ▪ *Share mutual appreciation between caregivers (I)* ▪ *Share responsibility to provide good care for client (F)* ▪ *Bring relevant quality themes under attention of caregivers (P)*

Mentioned by *C* Clients, *I* Informal caregivers, *F* formal caregivers, *P* managers/policy officers

Some participants viewed evaluating as complaining, preventing them from initiating an evaluation and preventing clients from honestly sharing care experiences, often leading to socially desirable answers. Difficulties in motivating formal caregivers to incorporate evaluations in the daily care process were mentioned, as were feelings that most quality evaluations are done twice without a clear reason. This lack of motivation from formal caregivers was also related to being on their own in the home care setting. The communication and collaboration opportunities between formal caregivers is perceived as low, especially for formal caregivers coming from an institutional long-term care setting. For instance, one formal caregiver, in relation to evaluating experienced QoC, mentioned that “*in home care, everyone is, yes you are alone [as a caregiver]. That is very different [than in a nursing home]. A lot relies on independence*.”

Evaluating care is perceived as a positive aspect in the care process that involves clients in the care process. It is seen as a moment where understanding for others and wishes can be shared. Talking about experiences also helped participants to recall relevant care experiences and communicate unfulfilled care needs*.* Desired gains related to how to speak more openly such as evaluating anonymously*.* One manager indicated that evaluation should be made “*anonymously, so you can get a lot more out of it than what clients might dare to say in person … I do not need to know which clients said that, because it will probably be something that several clients or informal caregivers have said*.”

Furthermore, care teams strive for shared ownership of an evaluation method in which mutual appreciation between caregivers is shared. For example, one district manager said that the measurement should be “*about being part of the team … because it will be as if I am imposing something [as a manager] and they have to do something with it*.” Bringing relevant quality themes under the attention of caregivers while evaluating QoC is believed to motivate incorporating more evaluations in daily practice.

#### What to do with the outcomes

When considering what to do with the outcomes while evaluating QoC (see Table 7 for an overview), there is a need to formulate concrete feedback and points-of-improvement, to avoid the use of difficult jargon while discussing evaluation outcomes, and to strive for outcomes that clearly reflect the evaluation content. Clear communication was also related to forming realistic expectations by clients based on the discussed content and evaluation outcomes. There was an effort to focus more on relevant themes that are within the scope of experienced quality of home care and to make room for discussing evaluation outcomes more extensively in care teams.

**Table 7:** Identified pains and (desired) gains for ‘what to do with outcomes’

Category	Pains	Gains
What to do with outcomes	▪ Little is done with outcomes, not transparent (I,F,P) ▪ No concrete points-of-improvement are being formed (I,F,P) ▪ Difficult jargon used in discussing outcomes of measurement (I) ▪ Outcomes do not reflect content evaluation (I) ▪ Outcomes only discussed by district nurse with direct caregiver (F) ▪ Unrealistic expectations of outcomes (F) ▪ Unrelated outcomes to nursing or personal care services (F) ▪ Only extreme outcomes are communicated in teams (P)	▪ Provide client insight into evaluation with both verbal and written outcomes (I) ▪ Discover specific points-of-attention for client (I) ▪ Discuss outcomes of evaluation with district nurses (F) ▪ Evaluation not aimed to solve all care difficulties, but rather to discuss them (F) ***Desired gains*** ▪ *Help stimulate caregiver to reflect on care provision (F,P)* ▪ *Discuss outcome evaluation in team and decide what to feedback to client (I,P)* ▪ *Share outcomes in team to define point-of-actions together (F, P)* ▪ *Help caregiver to check for unrecognized assumptions or biases in care process (F)* ▪ *Give daily update on positive and negative experiences in team (F)* ▪ *Access client file and outcomes remotely (F)* ▪ *Create client awareness for organizational restrictions on care services (F)* ▪ *Share outcomes evaluation in organization in multiple formats (*e.g. *figures and practical solutions) (P)*

Mentioned by *C* Clients, *I* Informal caregivers, *F* formal caregivers, *P* managers/policy officers

Participants mentioned pains with current evaluation methods were that they did not provide defined points-of-improvement and offered too little concrete feedback. An informal caregiver mentioned difficult jargon in discussing evaluation outcomes (e.g. care is being extended) and differences between evaluation content and what was written down afterwards, possibly contributing to undesired care outcomes. Forming unrealistic expectations for the client based on evaluation outcomes (e.g. adjust care planning) and discussing outcomes which are unrelated to nursing or personal care services (e.g. help with domestic chores) were highlighted by formal caregivers. Lastly, it was mentioned that only extreme outcomes are now being discussed in care teams and the rest is kept between the district nurse and direct caregiver.

Some informal caregivers mentioned that gains were that both verbal and written evaluation outcomes helped clients to gain insight in the care process and supported the discovery of specific points-of-attention. Formal caregivers mentioned that evaluation outcomes are currently being discussed with the district nurse; however, the aim is to discuss care difficulties but not solve them. Desired gains were that outcomes can help caregivers to reflect on their care provision and check for possible unrecognized assumptions or biases in the care process. It was mentioned that care teams should be the place where experiences are shared, outcomes evaluated, and together determine points-of-actions.

#### Prerequisites for evaluating

A number of prerequisites for evaluating experienced QoC were discovered (see Table 8 for an overview). Current pains related to a lack of communication (skills) among caregivers and the omission of both space and culture to discuss experiences in care teams. Relating to the team’s atmosphere, one formal caregiver mentioned that “*currently, I do not experience safety to discuss client experiences within my care team*.” The low literacy rate and self-reflection skills of clients makes it difficult to use standard evaluation methods such as questionnaires. Lastly, the cost related to evaluating was highlighted by policy officers. The desired gains in prerequisites related to stimulating a supportive atmosphere in care teams that allows for evaluations. Furthermore, it was desired that formal caregivers gain support through individual coaching to foster professionalism and develop skills both in conducting conversations and writing effective reports based on evaluations outcomes.

**Table 8:** Identified pains and (desired) gains for ‘prerequisites for evaluating’

Category	Pains	Gains
Prerequisites for evaluations	▪ Bad communication between caregivers in home care teams (F) ▪ No space and culture to discuss client experiences in team (F) ▪ Low literacy and self-reflection skills of clients (F) ▪ No evaluation moment because of costs (P) ▪ Missing communication skills evaluator (P)	***Desired gains:*** ▪ Individual coaching and support in team to conduct evaluations (I) ▪ Create supportive atmosphere in team (P) ▪ Foster professionalism and skills to conduct conversations (P) ▪ Gain skills in writing outcomes evaluation reports (P)

Mentioned by *C* Clients, *I* Informal caregivers, *F* formal caregivers, *P* managers/policy officers

## Discussion

The study discovered needs in measuring experienced QoC that resulted in an overview of goals, pains, and gains from key-stakeholders regarding the current methods used to evaluate experienced quality of home care in the Netherlands. The different goals in measuring experienced QoC were recognized by most key-stakeholders, who primarily related the goals to understanding and improving the primary care process of individual clients and secondarily to learning and improving the performance of home care teams. Six categories throughout the process of evaluating experienced QoC emerged in this study, namely: when to evaluate, who should evaluate, how to evaluate, what motivates one to evaluate, and what to do with the outcomes. In addition, prerequisites for evaluations were identified such as the importance for home care teams to foster communication skills and individual coaching and to create a supportive atmosphere for evaluating experienced QoC and using the outcomes for quality improvements.

By reflecting on the discovered needs (goals, pains, and gains) in evaluating experienced QoC a number of dilemmas came to light. First, it was not clear when to evaluate. This varied from constant evaluations as part of the care process to being initiated as needed by the client or formal caregiver to once every pre-set period (e.g. once every 6 months). A second dilemma that emerged is not knowing who is best as an evaluator to speak openly about experienced QoC. This varied from a direct caregiver having a (longstanding) care relationship with the client and knowing how to interpret their response during evaluations to a coordinating person within care teams who can act based on the evaluation outcomes to an external evaluator with sufficient care expertise to even a non-personal digital format (e.g. mail, website) to address difficulties anonymously. A third dilemma related to the structure in discussing and acting on evaluation outcomes. This varied from implicitly using outcomes to reflect upon one’s own care provision and individually checking for any unrecognized assumptions or biases in the care process to discussing evaluations outcomes within care teams to form concrete points-of-improvement to being explicit toward clients and informal caregivers about evaluation outcomes.

These dilemmas make it clear that it is important to define the QoC goal to be measured in home care before selecting and implementing a measure. It is known that different stakeholders in care organizations can have different (implicit) reasons for measuring experienced QoC [37]. This is also stated in existing models for selecting and implementing new measures, like the PROM cycle, which starts by defining the goal of a measure in the implementation process [20]. It is known that stating a clearly defined and achievable goal can help to feel motivated and committed in working toward that goal [38]. In determining when to evaluate, it is expected that experienced QoC should be measured more regularly when the goal is to gain insight and improve the primary care process, and less regularly when care teams and organizations are striving for an improvement on an overall level. A more continuous measurement of experienced QoC is also supported by the INDEXQUAL framework, defining it as a process before, during and after care is provided [15]. Furthermore, when deciding who is best as an evaluator, it should be clear which aspect of the care process will be discussed. For instance, one can wonder how clients perceive their direct caregiver as an evaluator when care experiences are being discussed involving that same caregiver. It needs to be considered that clients and informal caregivers are, to some extent, dependent on formal care providers, which can contribute to a fear of possible consequences when being completely honest about their experienced QoC [39]. However, care experiences on a team or organizational level, such as planning or access to care facilities, are some distance from the primary care process and can therefore be more easily discussed with direct caregivers. This highlights the importance of positioning the determined goal and individual needs within the different settings in home care before selecting a measure that is feasible for key-stakeholders, in line with the care process, as well as incorporating relevant experienced QoC attributes.

The results in this study underline the relevance of discussing care experiences during conversations imbedded in the care process above using questionnaires, which is in line with the trend of adopting more narrative evaluation methods [16, 40, 41]. This also depicts the dichotomy with existing quantitative quality measures, such as the Consumer Quality Index (CQ-index) and Net Promotor Score (NPS) [42]. These measures often focus on satisfaction as the main outcome, which has been shown to be an incomplete measure for experienced QoC as it generates gratitude bias and interpretation difficulties for formal caregivers [16, 42, 43]. In a previous study, attributes of experienced QoC were identified throughout the home care process [44]. These attributes include the presence of more ‘close’ personal care relationships (relating to trust, openness, and empathy) and the importance of care routines that are consistent with the client’s former way of living. Evaluating experienced QoC attributes during conversations provides valuable in-depth information on experienced QoC, for which standardized measures are insufficient [45, 46]. In order to select and implement a method to facilitate these conversations, a careful evaluation of suitable instruments and processes of how they will be used in organizations has to be designed in close collaboration with key-stakeholders [23, 47, 48]. This is also supported by the VPC, in which a value-proposition is made to relieve the identified pains and enhance the discovered gains [27]. One can determine which instrument’s features and which subsequent instrument are of greatest value to achieve a problem-solution fit. By adopting a research method such as the Participatory Action Research (PAR), stakeholders can thoroughly be involved in the following phase by carefully planning actions, reflections, and revision in short iterative cycles [49]. By doing so, the method that will best fit the needs for experienced QoC can be determined.

### Strengths and limitations

A strength in this study is that triangulation was used by combining both individual and group interviews, involving multiple perspectives. This made it possible for stakeholders from all layers of the home care setting to reflect on the questions brought forward. In qualitative research, involving multiple perspectives from different stakeholders is likely to result in an increased understanding of complex phenomena such as experienced QoC [50]. Individual interviews made it possible for clients and informal caregivers to receive extended information on examples for possible evaluation methods or visual stimuli to support them in formulating a response. For the individual interviews with clients and their informal caregivers, recruitment was done by their district nurses. This could have results in a selection bias such as only including clients with a less complex relationship or a specific client’s residence (sheltered housing estate). To account for this, other district nurses were asked to recruit clients, striving a balance regarding less complex versus more complex relationships or situations, presence of an informal caregiver (spouse or other) and the client’s residence (living in the community or sheltered housing estate). The focus group interviews allowed participants to respond to each other’s responses, generating a more thorough discussion of the topics compared to individual interviews. A disadvantage of using different methods with different stakeholders is that it was more challenging to analyze and compare the collected qualitative data. To overcome this challenge, we used the principles of the VPC to identify, structure, and analyze the individual needs for each group of key-stakeholders. However, it was decided not the present the findings as individual customer profiles, since it is believed that all key-stakeholder needs should be taken into account when determining which solution has the most value for the direct care process and organization [21, 22]. Furthermore, this research took place on a small scale in three care organizations focused on four key-stakeholders. It was therefore unclear whether data saturation was reached. Also, since clients included were aged above 75, it is unclear whether the findings in this study are applicable to clients populations below 75 or 65 years.

## Conclusions

This study indicates that home care organizations should consider selecting methods that fit to clients and caregivers’ needs and prevent dilemmas in evaluating experienced QoC. It is important to clearly define and communicate the goal of experienced QoC measures with all key-stakeholders and embed a feasible method in both the primary care process and care teams. Prerequisites for successfully assessing experienced QoC are that evaluators should have good communication skills, clients with low literacy and self-reflection skills should be able to sufficiently participate and feel heard during evaluations and a climate should be established in care teams to discuss evaluation outcomes. Formal caregivers in care teams should feel ownership over experienced QoC methods, so they are motivated to naturally incorporate it in the home care process. Clients and informal caregivers should feel supported to openly share care experiences with an evaluator, thereby being transparent about outcomes. Additionally, evaluations should lead to concrete points-of-improvement for the care process, avoid using difficult jargon while discussing evaluation outcomes, and strive for outcomes that clearly reflect the evaluation content. The findings of this study can serve as basis to develop or select methods, in co-creation with key-stakeholders, to assess the experienced quality in home care.

## Supplementary Information

**Additional file 1.**

## Data Availability

The datasets used and/or analyzed during the current study are available from the corresponding author on reasonable request.
