# Supplementary material for: The needs of key-stakeholders for evaluating client’s experienced quality of home care: a qualitative approach
Source: J Patient Rep Outcomes. 2020 Nov 10;4:96. doi: 10.1186/s41687-020-00260-3 (PMC7652985; doi:10.1186/s41687-020-00260-3)
Supplement: Supplementary file 1 — Additional file 1. [file 41687_2020_260_MOESM1_ESM.docx]

# Additional file 1

Example questions asked during individual interviews.

- What is your opinion toward measuring experienced quality in home care during the care process?
- Do current evaluation conversations give insight into the client’s experienced quality?
- In what situation is measuring experienced quality most desirable for clients?
- What is the client’s family perspective toward this?
- If we look at different examples of how to gain insight into experienced quality from the client’s perspective, what would be your opinion toward these methods?

Example questions asked during focus group interviews.

- If you look at different methods of how experienced quality can be evaluated, do you recognize these methods in the daily care process? Does anyone have experience with one of these methods?
- What do you find beneficial regarding the current way experienced care is evaluated?
- What do you find challenging or sub-optimal regarding the current way experienced care is evaluated?
- In your opinion, how are the outcomes of evaluations currently used?

Alternative questions for clients and informal caregivers during focus group interviews.

- Why do you want to discuss the care provided? What do you hope to achieve when discussing your care? What would you prefer to discuss about the care provided?
- What makes it difficult/challenging to discuss the care provided?
- What makes it easy/beneficial to discuss the care provided?
